# Effects of Critical Operation and Cleaning Parameters on Performances and Economic Benefits of Biogas Slurry Concentration by Forward Osmosis Membrane

**DOI:** 10.3390/membranes13030288

**Published:** 2023-02-28

**Authors:** Bangxi Zhang, Tianhong Fu, Qinyu Zhang, Xiaomin Wang, Ling Tang, Quanquan Wei, Yun Li, Yutao Peng

**Affiliations:** 1 Institute of Agricultural Resources and Environment, Guizhou Academy of Agricultural Sciences, Guiyang 550006, China; 2 School of Pharmacy, Zunyi Medical University, Zunyi 563006, China; 3 College of Resource and Environment, Qingdao Agricultural University, Qingdao 266109, China; 4 School of Agriculture, Sun Yat-Sen University, Shenzhen 518107, China

**Keywords:** biogas slurry, forward osmosis, membrane fouling, optimization, membrane cleaning

## Abstract

Forward osmosis membrane technology (FO) shows potential application prospects in biogas slurry concentration, which is conducive to promoting the sustainable development of biogas projects. However, at present, the key influencing factors of membrane concentration using FO are not well understood. Therefore, this study analyzed the influence of draw solution concentration, pH, temperature and cross-flow velocity on the concentration efficiency of FO membrane, and optimized the operation parameters of FO membrane. The results showed that the concentration effect of the NaCl draw solution at pH 5 or 9 was better than that at pH 7. The order of factor influencing the water flux was as follows: draw liquid concentration > cross-flow velocity > operating temperature. The optimal combination obtained by orthogonal analysis was under 45 °C, with a cross-flow velocity of 1 L/min and the use of 1.5 mol/L NaCl as draw solution. The results of the membrane cleaning implied that the recovery rate of the fouled membrane after acid–base cleaning is significantly higher (88%) than other cleaning solutions. This research offers a scientific reference for applying positive osmosis technology to re-utilize biogas slurry resources.

## 1. Introduction

Due to the rapid population growth, people’s dependence on energy is increasing daily. Fossil fuel depletion and accompanying pollution raise serious concerns [1]. Therefore, a large number of researchers focus on the field of sustainable and clean energy [2]. Biogas is a renewable and clean energy that plays an increasingly important role in energy production and environmental protection [3]. In the process of biogas production, a substantial amount of biogas slurry with rich organic matter and nutrients will be generated, which can be used as organic fertilizer [4]. However, biogas slurry has a large volume and has high storage and transportation costs, which limit the biogas slurry serving as a fertilizer for direct irrigation [5]. In addition, the absorptive capacity of the soil is much lower than the capacity of biogas slurry production [6]. Therefore, a considerable amount of biogas slurry is still directly discharged because it cannot be used in time, which not only wastes resources but also poses a threat to the environment and human health [7].

Concentrating and recycling biogas slurry is the fundamental way to solve the above-mentioned problems [8]. In biogas slurry concentration technology, membrane separation technology has become an ideal choice for biogas slurry concentration due to its cost-effective and convenient operation and no change in composition characteristics. Ultrafiltration, nanofiltration and reverse osmosis technology are the main technologies [5,9,10]. The concentrated liquid has the characteristics of unchanged nutrients, small volume, high concentration, easy transportation and storage, etc. [5]. However, the membrane separation process usually leads to severe membrane fouling as a large amount of suspended particles, organic matter, colloidal particles and inorganic matter is contained in biogas slurry [11]. Therefore, the development of membrane separation with low fouling degree is very important to improve the concentration efficiency of biogas slurry. The forward osmosis (FO) membrane separation process is an emerging membrane separation technology, which has the characteristics of high selectivity, low pollution tendency, high pollution reversibility and low energy consumption in proper treatment [12]. Therefore, FO membrane technology has shown good application prospects for biogas slurry concentration.

However, the FO membrane separation process is affected by many factors, including the type of draw solution, pH, cross-flow velocity and temperature [13]. In addition, in the process of biogas slurry concentration, the fouling of the FO membrane will reduce its concentration efficiency. Herein, recovery of the FO membrane with simple method is beneficial to reduce the cost of FO technology for biogas slurry concentration [14]. The filtration efficiency of the entire FO membrane system can be improved by optimizing the operating parameters; the membrane flux of the fouled membrane can be restored by membrane cleaning and prolonging the service life of the membrane [15]. The commonly used cleaning methods include physical cleaning and chemical cleaning. Physical cleaning mainly includes ultrasonic cleaning, water backwashing, air backwashing and sponge ball cleaning [16]. In some cases, they can improve cleaning efficiency, but do not completely remove membrane fouling [17]. Therefore, diverse chemical cleaning methodologies have been studied. Chemical cleaning refers to the removal of contaminants by soaking the membrane in a cleaning solvent and then rinsing [18]. Commonly used chemical cleaning agents for membrane fouling include acids (HCl, HNO_3_, H_3_PO_4_, citric acid, etc.), alkalis (NaOH, KOH, etc.), oxidants, surfactants, enzymes and chelating agents. [16]. According to the different nature of membrane fouling, the cleaning agent used in membrane fouling cleaning is also different [17]. For example, acidic cleaning draw solution can be used to clear up inorganic salts such as calcium carbonate on the membrane surface [19], and alkaline cleaning agents should be used to remove organic matter on the membrane surface [20]. In addition, some scholars have combined physical and chemical methods to clean the fouling membrane, which can almost return to the original flux after cleaning [21]. Generally, chemical agents are used to clean the membrane regularly to maintain the membrane flux. However, the cleaning frequency will greatly affect the membrane fouling and service life [22]. In general, for the concentration of biogas slurry treated by FO membrane, it is necessary to optimize the FO concentration process parameters and clean the fouled membrane to improve the concentration efficiency of the system and control the membrane fouling. However, there are few studies on the optimization of each influencing parameter and the effect of different cleaning methods in the FO membrane cleaning process. More effort is needed to explore the effects of different influencing factors and membrane cleaning methods on the FO concentration effect in biogas slurry treatment.

In the previous study, we investigated the performance of different types of draw solution in biogas slurry FO concentration, and found that NaCl has the characteristics of good concentration effect, low cost and easy availability [13]. Therefore, NaCl was selected as the draw solution in this study. First, on the basis of exploring the effect of draw solution pH on the concentration of biogas slurry in FO membrane, this study investigated the effects of different temperatures, cross-flow velocity and concentration of draw solution through orthogonal experiments. Secondly, in view of the fouling of FO membrane in the biogas slurry concentration process, this study selected physical membrane cleaning technologies including osmotic reflux, pressure reflux and cross-flow flushing and acid–base cleaning chemical technologies to study the impact of membrane cleaning on the FO concentration effect. It provides a theoretical basis for promoting the re-utilization of biogas slurry, reducing resource waste and improving the efficiency of biogas slurry concentration by FO method, and optimizing the process parameters of biogas slurry FO concentration and membrane cleaning to effectively control membrane pollution.

## 2. Materials and Methods

### 2.1. Experiment Materials

The biogas slurry was collected from a local pig farm (Runtian farmyard, Qingdao, China). The collected biogas slurry is the supernatant after natural sedimentation in the temporary storage tank. The basic physical and chemical properties of the biogas slurry were as follows: pH 8.02, chemical oxygen demand (COD) 312 mg/L, total nitrogen (TN) 412 mg/L, total phosphorus (TP) 28.5 mg/L, NH_3_-N 152 mg/L, NO_3_-N 38.6 mg/L, and PO_4_^3−^-P 35.6 mg/kg. Traditional polyamide membranes (Hydration technology innovations, Albany, OR, USA) were used in this study, which consist of a thin selective polyamide active layer on top of a porous polysulfone support layer [11].

### 2.2. FO System

**Figure 1:**
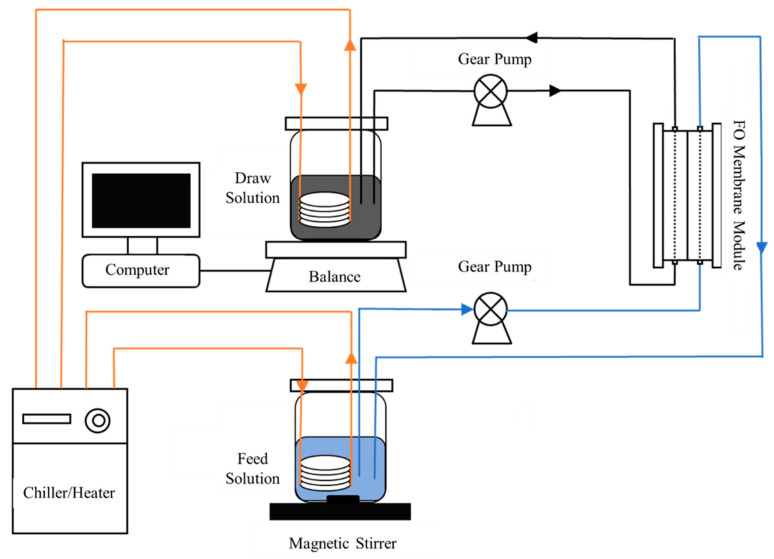
The diagram using forward osmosis membrane technology for biogas slurry concentration.

The concentration of biogas slurry was conducted with a bench scale FO system with a cross-flow membrane cell and two variable speed gear pumps (Figure 1). The membrane cell was composed of two separated acrylic blocks to hold a flat-sheet membrane without any physical support. The draw solution reservoir was placed on a digital balance which is connected to a computer automatically recording weight changes and calculating permeate water flux.

### 2.3. Influence Test of Different Process Parameters

In our previous optimization study (data not given in this study), 0.5 mol/L NaCl solution showed the best performance of the biogas slurry concentration. In this research, this 0.5 mol/L NaCl solution was further adopted to optimize the pH effects of draw solution. The FO system was operated in osmotic dilution mode with biogas slurry as the feed solution. Weigh 14.6 g of solid sodium chloride with an analytical balance, dissolve it in distilled water and dilute to 500 mL to prepare a 0.5 mol L^−1^ NaCl draw solution. The pH of the prepared NaCl solution was measured with a pH meter, and the pH of the draw solution was adjusted to pH = 5, pH = 7 and pH = 9 with 1 mol L^−1^ HCl and NaOH, respectively. NaCl solutions with different pH values were used as the draw solution. The initial volume of the feed and draw solutions was 500 mL. The membrane active layer faces the feed solution. Each experiment was terminated when the observed water flux decreased to a negligible level. During the FO operation, aqueous samples (5 mL) were extracted from the feed and extraction solutions at intervals. All experiments were repeated with new membrane samples. The most suitable draw solution was screened out according to the water recovery rate.

On the basis of the influence test of the pH of the draw solution on the concentration of FO, a three-factor and three-level test of the concentration of the draw solution, temperature and cross-flow velocity was carried out. The orthogonal test design is shown in Table 1, and the test conditions are consistent with the pH test of the draw solution.

### 2.4. Membrane Cleaning Test

The FO membrane after the concentration processes was rinsed, and then osmotic backwash was performed to evaluate the reversibility of membrane fouling. This is the same as permeate backflow, and the flow rate was increased to 2 times, which is pressure permeation cleaning. The flushing method involves exchanging the flow of the feed liquid and the drawn liquid to flush the membrane for 30 min, that is, cross-flow cleaning. An amount of 0.1 mol/L oxalic acid was used for acid washing, and 0.1 mol/L NaOH solution was used for alkali washing. The pure water membrane flux was measured, including the pure water flux of the FO membrane after fouling, after flushing, after osmosis backwashing, after acid washing and alkali washing. The FO system was run for 2 h and the average pure water flux was obtained using a 1 L deionized water feed and a 1 M NaCl draw solution The pure water flux recovery (ŋ) after membrane cleaning is calculated as [23]:

(1) $$\eta \left(\%\right)=\frac{J_{c}-J_{a}}{J_{b}-J_{a}}\times 100$$

where J_b_ and J_a_ are the pure water fluxes before and after biogas slurry concentration, respectively; J_c_ is the pure water flux after membrane cleaning. The water flux recovery rate indicates the reversible nature of membrane fouling.

### 2.5. Analytic Methods

The three-dimensional morphology of the membrane surface was observed using atomic force microscopy (AFM, Nanoman, Billerica, MA, USA) [11]. Scanning electron microscope (SEM) combined with energy dispersive spectrometer (EDS) (JCM-6000, JEOL, Tokyo, Japan) was utilized to observe the morphological and compositional characteristics of the membrane surface. Membrane surface functional groups were characterized by attenuated total reflection Fourier transform infrared (ATR-FTIR) spectroscopy (IRAffinity-1, Shimadzu, Kyoto, Japan) [24].

## 3. Results and Discussion

### 3.1. FO Operation Parameters Optimization

#### 3.1.1. Influence of pH Value of Draw Solution

**Figure 2:**
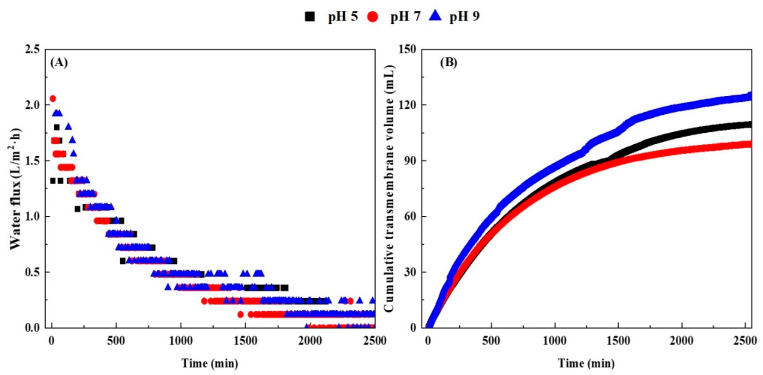
The effect of the draw solution (0.5 mol/L NaCl) pH on the water flux in the concentration process of the biogas slurry FO membrane (**A**), and the effect of the draw solution pH on the cumulative transmembrane volume (**B**).

Figure 2A shows the effect of different pHs of the draw solution on the average water flux. Some studies believe that the concentration polarization inside the biogas slurry is the key factor affecting the water flux. Concentration polarization means that during the operation of the forward osmosis membrane, the concentrated elements and organic matter are blocked near the membrane, and the concentration is greater than that of the biogas slurry. Water molecules flow from the high concentration side to the low concentration side, resulting in a decrease in water flux [25,26], while the blockage of the forward osmosis membrane by various substances in the biogas slurry is also an important reason for the decrease in the average water flux. As the operating time continues to increase, more substances will accumulate on the membrane, blocking the forward osmosis membrane and resulting in a decrease in water flux [27]. As shown in Figure 2A, in which a 0.5 mol/L NaCl solution was chosen as the extraction solution, the osmotic driving force at pH = 9 and the average water flux was the largest. The average water flux of the draw solution with pH = 7 was the smallest. The draw solution with pH = 9 is more conducive to the recovery of water in the biogas slurry (Figure 2).

The cumulative transmembrane volume refers to the concentration difference of the solution on both sides of the forward osmosis membrane, which generates osmotic pressure, and the water molecules move from the side with low osmotic pressure to the side with high osmotic pressure, until the volume of water passing through when the osmotic pressure of the solution on both sides is equal. It is obvious from Figure 2B that the pH of the draw solution has a great influence on the volume of water molecules permeating through the FO membrane. The draw solution with pH = 9 was more favorable for water molecules to pass through the FO membrane, and the transmembrane volume reached 125.20 mL at 40 h. The cumulative transmembrane volume of draw solution with pH = 5 is less than that of draw solution with pH = 9, reaching 110.40 mL at 40 h. The cumulative transmembrane volume of water molecules under the action of the draw solution with pH = 7 was the lowest, reaching 99.90 mL at 40 h.

**Figure 3:**
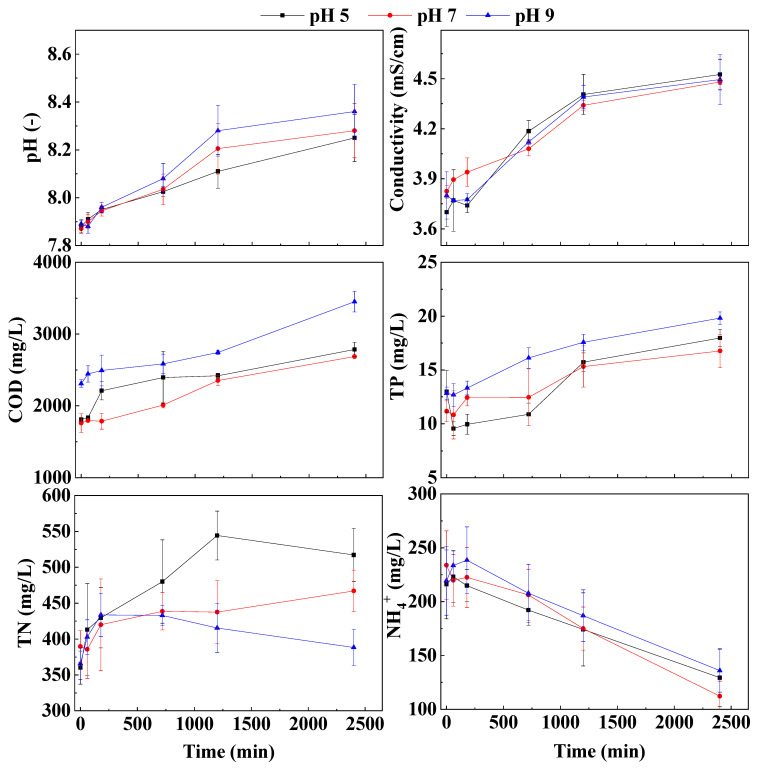
Effect of draw solution pH on basic properties and nutrients of biogas slurry during FO membrane concentration processes.

As shown in Figure 3, when the draw solution pH is 5, the pH of the biogas slurry increases most significantly, and the pH value rises from 7.89 to 8.36. When the pH of the draw liquid is 9, the pH of the biogas slurry rises less than the other two, from 7.88 to 8.25. The effect of the extraction liquid at pH = 7 can increase the biogas slurry pH from 7.87 to 8.28. Under the action of osmotic pressure, the extraction liquid draws a certain amount of water molecules from the biogas slurry. Afterwards, the reduction of water leads to a decrease in the amount of solvent in the biogas slurry [28]. It can be clearly seen from Figure 3 that the biogas slurry pH increased significantly under the action of the three different pH draw solutions. The initial osmotic pressure of the draw solution is higher than that of the original biogas slurry, and the conductivity of the draw solution is also higher. With the movement of water molecules, the concentration of the NaCl solution in the draw solution gradually decreased. On the contrary, the concentration of total phosphorus, COD and other indicators in the biogas slurry, and the conductivity increased accordingly. The conductivity increases for two reasons [28]. The natural salinity of the feed solution accumulates in the feed solution due to membrane retention and concentration. The reverse diffusion of draw solutes into the feed solution also contributes to salinity accumulation [29,30]. At 40 h, the conductivity of the extraction liquid and biogas slurry gradually increased and became stable, and the movement of water molecules on the forward osmosis also correspondingly decreased.

The concentration of total phosphorus in the biogas slurry gradually increased under the action of different pH draw solutions. It has been reported that FO membranes can almost completely retain phosphorus due to the large hydration radius of phosphate and its electrostatic repulsion to negatively charged membrane surfaces (Schneider et al., 2019). When the pH of the extraction solution was 9, the total phosphorus concentration of the biogas slurry was 64.2, 63.3, 66.6, 80.6, 87.8 and 99.1 mg/L at 0 h, 1 h, 3 h, 12 h, 20 h and 40 h, respectively. The experimental results showed that the total phosphorus concentration at the beginning is greater than that of the biogas slurry after the operation. The main reason for this phenomenon is that the biogas slurry sampled at 0 h is not concentrated. As shown in Figure 3, when the NaCl solution of pH = 9 is used as the draw solution, the increased rate of the concentration of total phosphorus and the final concentration are significantly greater than those of the NaCl draw solution of pH = 5 and pH = 7. Before 20 h, the concentration rate of total phosphorus in NaCl solution at pH = 7 was significantly higher than that in NaCl solution at pH = 5. After 20 h, the concentration rate of total phosphorus in biogas slurry by NaCl solution with pH = 5 was higher than that of NaCl solution with pH = 7, and the concentration degree at the final 40 h was also higher than that of NaCl solution with pH = 7. It can be seen that the extraction solution with pH = 9 is more conducive to the concentration of total phosphorus. The COD concentration in the biogas slurry gradually increased under the action of the extraction solution with different pHs. Regarding the effect of different pH draw solutions on the concentration effect of COD in biogas slurry, the concentration effect of the NaCl draw solution with pH = 9 is the best. Since the biogas slurry was placed in a beaker without sealing treatment during the experiment, the ammonia nitrogen in the biogas slurry is easily volatile, so the concentration of ammonia-nitrogen will gradually decrease [31]. In addition, due to the smaller hydration radius of nitrogen and its high diffusivity, it also leads to the low rejection of nitrogen (especially NH_4_^+^-N) by the FO membrane (Arena et al., 2014; Schneider et al., 2019).

Overall, the concentration effect when the pH of the draw solution is 5 or 9 is better than that when it is neutral. However, considering the cost of the pH adjustment process, it is recommended to adjust the pH of the draw solution when necessary.

#### 3.1.2. Effects of Draw Solution Concentration, Cross-Flow Velocity and Temperature

**Figure 4:**
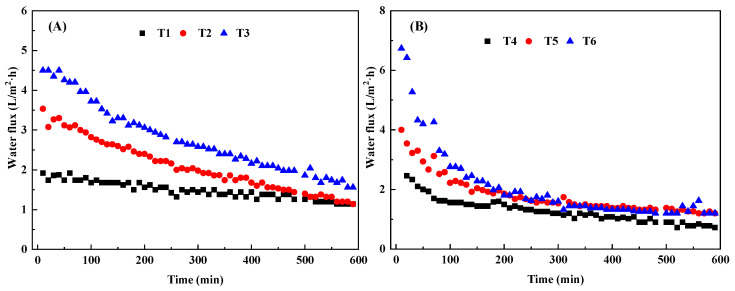

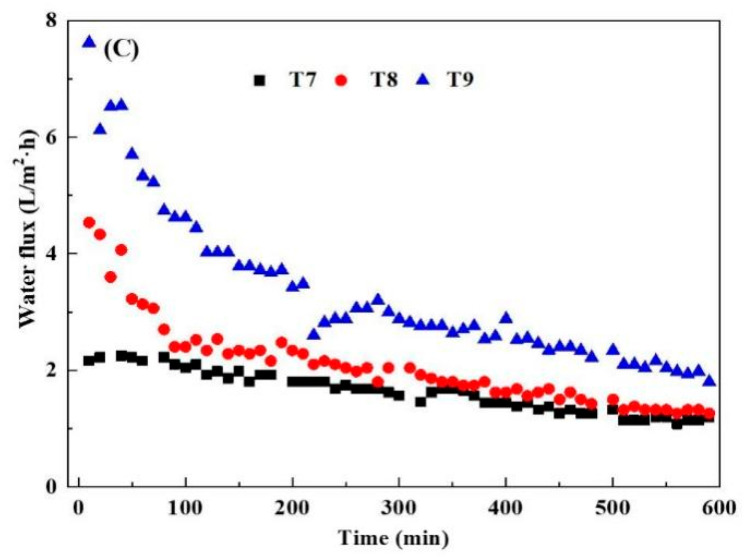
Change in water flux in orthogonal experiment of biogas slurry FO membrane concentration (T1, T2, T3) (**A**), (T4, T5, T6) (**B**), (T7, T8, T9) (**C**).

It can be seen from Figure 4A that the fluxes treated by T1-T3 all showed a decreasing trend. Since each treatment was carried out at 15 °C, the concentration of the draw solution and the cross-flow velocity were the influencing factors of the water flux difference between the different treatments. The initial water flux of T3 treatment was the highest, reaching 4.5 L/m^2^·h, while the initial water flux of T1 treatment was the lowest, only 2 L/m^2^·h. The initial water flux of T3 treatment was 55% higher than that of T1 treatment, because T1 treatment had the lowest draw solution concentration and the lowest cross-flow velocity. Therefore, it can be judged from Figure 3A that the constant temperature can result in the higher concentration of the draw solution and the greater the cross-flow velocity, the higher the water flux [32,33]. When the concentration of biogas slurry FO membrane lasted for 600 min, the fluxes of T2 and T1 treatment water flux were decreased to about 1.5 L/m^2^·h. However, the water flux of T3 treatment at 600 min was still 25% higher than that of the other two treatments due to the high concentration of draw solution and high cross-flow velocity of T3 treatment.

As can be seen from Figure 4B that at 25 °C, the water flux of each treatment experienced a trend of first rapidly declining and then gradually smoothing. As shown in Figure 4A, it is obvious that the temperature increases exerted a positive effect on the initial water flux of each treatment. This is because temperature increased the diffusion rate of water molecules and solutes in the draw solution, and water flux will increase with the increased temperature [15]. T6 treatment had the highest initial water flux, which reached 6.8 L/m^2^·h, while T4 treatment had the lowest initial water flux, which was about 2.6 L/m^2^·h. Although the cross-flow velocity of T4 treatment is higher than that of T6 treatment, the initial water flux of T6 treatment is 62% higher than that of T4 treatment, which may be due to the lower concentration efficiency of draw solution in T4 treatment. Therefore, it can be inferred from these results that at constant temperature, the influence of the concentration of the draw solution on the water flux is greater than that of the cross-flow velocity. At the end of the FO membrane concentration test, the water fluxes of T5 and T6 treatments were dropped to 1.7 L/m^2^·h. However, due to the low concentration of the draw solution in the T4 treatment, the water flux of this treatment was always lower than the other two treatments. Since each treatment is carried out at the same temperature, and there is no corresponding relationship between the concentration of the draw solution and the cross-flow velocity, it is impossible to directly analyze the influencing factors of the difference in water flux between different treatments.

It can be seen from Figure 4c that the fluxes of T7-T9 treatments all showed the same trend as T4-T6 treatments, which first decreased and then leveled off. Compared with the experiments conducted at 15 °C and 25 °C, the test conducted at 45 °C had the highest initial water flux. The water flux of T9 treatment reached 7.7 L/m^2^·h. However, the initial water flux of T7 treatment was the lowest, which was only 2.2 L/m^2^·h. This may be due to the low concentration of the draw solution at the beginning of the treatment. The initial water flux of the T9 treatment was 70% higher than that of the T7 treatment, probably because the extraction liquid concentration of the T7 treatment was the lowest, and the high temperature led to a large difference in the water flux. When the FO membrane was concentrated for about 10 h, the water flux of T9 treatment was still about 2 L/m^2^·h. However, the water flux of the other two treatments has dropped to about 1.6 L/m^2^·h, which is only 80% of the final water flux of the T9 treatment. Since there is no corresponding relationship between the concentration of the draw solution and the cross-flow velocity in each treatment, it needs to be further explored through orthogonal analysis.

#### 3.1.3. Correlation Analysis

It can be seen from the orthogonal analysis (Table 2) that the influence of each factor on the water flux in the biogas slurry FO membrane concentration process is as follows: draw liquid concentration > cross-flow velocity > operating temperature. This is because the increase in draw solution concentration provides higher ion content for the FO membrane concentration system, thus providing a stronger driving force for the FO membrane concentration system to achieve the highest water flux [34]. The operating temperature also has a positive effect on the water flux in the biogas slurry FO membrane concentration process, but it is less significant than the improvement effect of the draw solution concentration and cross-flow velocity on the water flux. Temperature control consumes more energy and is more difficult to achieve. In addition, the results of the orthogonal variance significance analysis also showed that at the level of α = 0.05, the concentration of the draw solution had a significant effect on the change in the water flux during the FO membrane concentration process of biogas slurry (F_B_ = 86.54 > 0.05) (Table 3). Previous research of Xu, Ye, Li, Song and Xiao [32] reported that the cross-flow velocity of 20.5 cm/s was considered to be preferred for biogas concentration during FO process. When the cross-flow velocity was increased to be 25.6 and 30.7 cm/s, the water flux declined quickly, which was possibly due to the more sever internal concentration polarization and membrane fouling caused by the higher initial water flux. However, at the level of α = 0.05, operating temperature and cross-flow velocity had no significant effect on the change in water flux during biogas slurry FO membrane concentration (F_A_ = 7.25 < 0.05(2, 2) = 19; F_C_ = 3.04 < 0.05), which indicated that the optimal combination obtained by orthogonal analysis was 45 °C, 1.5 mol/L NaCl as the draw solution and the 1 L/min of cross-flow velocity. Under the optimum conditions, the FO process can discharge enough water to concentrate the biogas slurry, and the concentrated biogas slurry can be well-concentrated liquid fertilizer [15,35].

### 3.2. Membrane Cleaning

#### 3.2.1. Effects of Membrane Fouling on Water Flux

**Figure 5:**
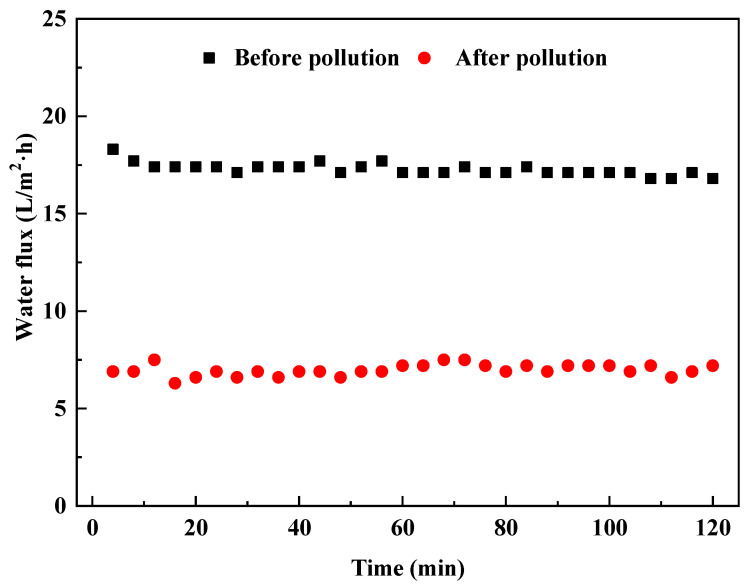
Changes in water flux before and after FO membrane fouling.

It can be seen from Figure 5 that after the biogas slurry is concentrated, the membrane fouling significantly hinders the flux of clean water through the membrane. Before membrane fouling, the FO membrane flux test was carried out with clean water, and the water flux of about 18 L/m^2^·h could be achieved, which proved that the FO membrane has a remarkable effect in the recovery of pure water. In the process of using the fouled membrane after the biogas slurry concentration to conduct the pure water FO membrane flux test, the water flux was around 7 L/m^2^·h, which was similar to Schneider et al. [36], which showed that FO water flux reached 3 L/m^2^·h with 0.66 M MgCl_2_ as the initial draw solution concentration. Our previous study showed that the viscous fouling layer aggregated by biogas slurry completely covered the surface of FO membrane after membrane concentration. A filter cake layer composed of macromolecular particles is formed on the surface [13]. The membrane fouling layer has the characteristics of rough surface and obvious large particles. In theory, the purpose of a large-scale recovery of membrane flux can be achieved by physical cleaning such as water washing [37]. In addition, infrared spectrum analysis and energy spectrum analysis also show that there may be organic substances such as proteins and polysaccharides in membrane pollutants, so the use of acid–base cleaning can play a certain role in removing organic substances [13].

#### 3.2.2. Cleaning Effect of Physical Cleaning Method

**Figure 6:**
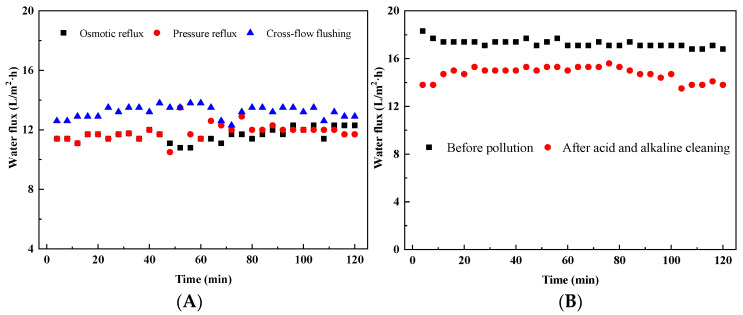
The effect of different physical cleaning methods on water flux (**A**), and the effect of acid and alkaline cleaning methods on water flux (**B**).

The fouled membrane produced by the concentration of biogas slurry FO membrane can reach a pure water flux of about 11.5 L/m^2^·h after osmotic reflux (Figure 6A). Compared with the pure water flux of the fouled membrane, this cleaning method can restore about 40% of the FO membrane flux. This may be because the permeate reflux takes away some of the pollutants in the membrane fouling layer that are not adhered to the FO membrane surface [38]. Pressure reflux cleaning increases the flow of cleaning water to a certain extent and takes away more contaminants on the surface of the FO membrane. However, the improvement of pure water flux is not significant, and the recovery rate of this cleaning method for FO membrane pure water flux is also about 40%. This may be due to the formation of a tight membrane fouling layer in the same direction as the water flow during the membrane concentration process. Flushing with water in the same direction can only take away non-adhered contaminants, implying that membrane fouling was fully reversible by physical flushing [12], but most contaminants are difficult to remove in this way.

Compared with the pressure reflux cleaning method, the cross-flow cleaning method significantly improves the pure water flux of the fouled membrane. The pure water flux of the fouled membrane after cross-flow cleaning can reach about 13 L/m^2^·h, which restores about 70% of the pure water flux of the FO membrane (Figure 6A). This is because after the cross flow, some pollutants that are difficult to be washed away by the osmotic reflux are washed away, and the further cleaning effect of the fouled membrane is realized.

#### 3.2.3. The Effect of Acid and Alkaline Cleaning

On the basis of physical cleaning, the use of acid and alkaline cleaning can effectively improve the FO membrane flux (Figure 6B). Acid and alkaline cleaning can remove calcium carbonate and organic matter in the fouling layer of FO membrane [39]. Due to the complex composition of biogas slurry, the composition of the FO membrane fouling layer includes organic and inorganic fouling [13]. Through physical cleaning, most of the membrane fouling caused by the membrane fouling layer can be removed. However, some pollutants, especially organic pollutants, are difficult to remove after being adsorbed on the FO membrane. More than 88% of the flux of the FO membrane can be recovered by acid washing and alkali washing. It is difficult to completely restore the original flux of the FO membrane by the membrane cleaning methods of acid washing and alkali washing. The main reason is that when the size of the feed molecule is smaller than the membrane pore size, the particles will sink into the membrane pores and cause irreversible pollution [40]. However, there is also evidence that the excessive use of chemical agents to regularly clean the membrane can maintain the membrane flux, but the cleaning frequency greatly affects the membrane fouling and service life [22].

#### 3.2.4. SEM-EDS Images of the Membrane before and after Cleaning

**Figure 7:**
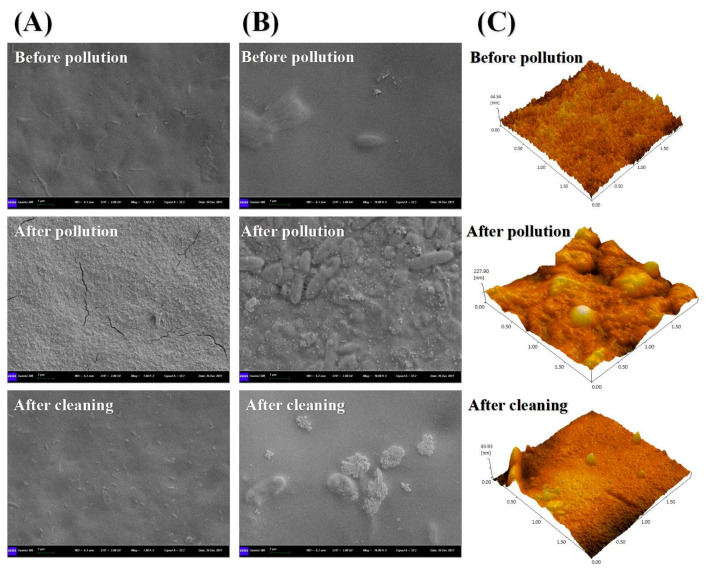
SEM images (5 um scale) (**A**), (1 um scale) (**B**), and AFM images (**C**) of FO membrane before pollution, after pollution and after cleaning.

As shown in Figure 7, after the membrane is cleaned, the contamination layer formed by the membrane fouling can be removed in a large area. Before the membrane fouling, the surface of the FO membrane was relatively smooth, and no impurities appeared except for part of the supporting skeleton. After the biogas slurry concentration test, a fouling layer will form on the surface of the FO membrane. Through membrane cleaning, it can be clearly seen that the fouling layer covering the surface of the FO membrane has been basically removed. However, although the FO membrane fouling layer has been removed after physical cleaning and acid–base cleaning, there are still some unremoved membrane pollutants on the surface of the FO membrane, which is also the main reason why the membrane flux cannot be fully recovered after membrane cleaning.

Compared with the SEM image of 5 μm size, the effect of membrane cleaning is better reflected in the 1 μm size (Figure 7). Under the 1 μm specification, it is obvious that after the membrane fouling layer is formed, the FO membrane is completely covered, and a rough appearance of the membrane fouling layer is formed, which not only affects the water flux but also more easily adsorbs more membrane pollutants. The surface of the new FO membrane is smoother than that of the contaminated and cleaned membranes. Membrane cleaning can remove most rod-shaped and block-shaped contaminants on the membrane surface, but some cluster-shaped contaminants remain.

#### 3.2.5. AFM Images before and after Membrane Cleaning

It can be seen from Figure 7C that the three-dimensional roughness of the membrane surface was significantly reduced after membrane cleaning, but it still did not return to the state of a new membrane. The initial FO membrane surface roughness was about 44 nm, and the membrane surface roughness was increased to 228 nm after membrane fouling. After membrane cleaning, the surface roughness of the FO membrane decreased by 64%. It is proven again that membrane cleaning can significantly reduce the membrane pollutants produced by the concentration of biogas slurry FO membrane. At the same time, this is also an intrinsic factor for membrane cleaning to significantly increase the water flux of FO membranes. In addition, it can be seen from the AFM image after membrane cleaning that some pollutants still exist on the membrane surface, which has not achieved the effect of complete cleaning. Due to the increased roughness of the membrane surface, reuse of the cleaned FO for biogas slurry concentration may enhance the adhesion of organic matter and microorganisms.

#### 3.2.6. Changes in Basic Properties before and after Membrane Cleaning

There is a significant difference in the salt permeability coefficients (i.e., A and B values) before and after membrane cleaning (Table 4). This difference can be attributed to the effect of the membrane fouling layer. In contrast, the membrane structure parameters (S values) of the two FO membranes did not change significantly. This may be because the membrane fouling layer, although significantly affecting the flux, does not damage the polymer structure on the membrane surface. The S value of the fouled membrane was slightly higher, indicating that its support layer was thicker and less porous than the cleaned FO membrane. In contrast, the A value of the FO membrane after cleaning is much higher. This observation indicates that cleaning the FO membrane after the membrane fouling layer can significantly improve its transport performance, thereby enhancing the performance of the water flux in the subsequent concentration process. At the same time, the B value of the FO membrane after cleaning is also about 80% higher than the corresponding value after membrane fouling, which indicates that the membrane fouling layer has the purpose of isolating pollutants to a certain extent.

#### 3.2.7. Economic Cost Analysis

One of the disadvantages of biogas slurry concentration is the cost. Compared with pressure-driven membranes, FO does not require external operating pressure, and the corresponding operating energy consumption and costs are lower, but membrane cleaning will also increase costs to a certain extent. As shown in Table 5, regardless of the investment cost of cleaning equipment, the cost of electricity generated by the unit membrane osmotic reflux flushing process is 10 yuan, and the water is 2 yuan. The pressure reflux requires double the water, electricity and other related costs. In the process of cross-flow flushing, due to the need to switch the interface, there is a certain water loss in the process, which is calculated by double the cost of water. The acid–base cleaning process will generate corresponding reagent costs, which are calculated at 5 yuan. About 40% of the FO membrane flux can be recovered by using osmotic reflux, and the cost is only 12 yuan per unit of FO membrane. Due to the limited degree of water flux improvement brought by pressure reflux, it is recommended to bypass the pressure reflux cleaning operation and directly perform the cross-flow operation in actual operation, and the cost per unit area is about 24 yuan per unit of FO membrane. After osmotic backflow and cross-flow cleaning, the FO membrane can recover 70% of the membrane flux, which basically meets the requirements for continued use, and can be used for subsequent biogas slurry membrane concentration applications. If there are special requirements for the flux of the FO membrane, the cost of 19 yuan per unit of FO membrane can be added to restore the FO membrane to 88% of the membrane flux of the new membrane. Since membrane module investment is the main factor affecting the total investment in the membrane concentration system, the cost of the membrane module accounts for about 5% to 30% of the cost of the membrane concentration system. Therefore, recycling the FO membrane through membrane cleaning technology is one of the ways to reduce the total cost.

## 4. Conclusions

The results of this study showed that optimizing the operating parameters can effectively improve the concentration efficiency of the FO system. The concentration effect when the pH of the draw solution is 5 or 9 is better than that when the pH is 7. The influence degree of each factor on the water flux in the biogas slurry concentration is as follows: draw solution concentration > cross-flow velocity > operating temperature. The optimal combination obtained by orthogonal analysis is under the condition of 45 °C, with 1.5 mol/L NaCl used as the draw solution and when the biogas slurry membrane concentration is carried out at a cross-flow velocity of 1 L/min. In addition, the removal effect of membrane cleaning on membrane fouling is very significant. The fouled membrane can recover about 40% of the FO membrane flux after osmosis reflux; after cross-flow flushing, the FO membrane can recover 70% of the membrane flux; after acid–base cleaning, a better flux recovery level can be obtained. Membrane cleaning can remove most of the rod-shaped and block-shaped pollutants on the membrane surface, but some cluster pollutants remain after cleaning, so it is difficult for membrane cleaning to restore the FO membrane to the flux level of a new membrane. Current cleaning process strategies are insufficient to achieve full flux recovery. In order to better understand the cleaning process, more in-depth research is required.

## Figures and Tables

**Table 1:** Orthogonal experimental design of FO membrane concentration.

Treatment	Temperature	Concentration	Cross-Flow Velocity
	(°C)	(mol L^−1^)	(L min^−1^)
T1	15	0.5	0.5
T2	15	1	1
T3	15	1.5	1.5
T4	25	0.5	1
T5	25	1	1.5
T6	25	1.5	0.5
T7	45	0.5	1.5
T8	45	1	0.5
T9	45	1.5	1

**Table 2:** Orthogonal analysis of FO membrane concentration.

Test Number	Factors			Water Flux (L/m^2^·h)
	Temperature (°C)	Draw Solution Concentration (mol/L)	Cross-Flow Speed (L/min)	
T1	15	0.5	0.5	1.84
T2	15	1.0	1.0	3.23
T3	15	1.5	1.5	4.39
T4	25	0.5	1.0	2.17
T5	25	1.0	1.5	3.28
T6	25	1.5	0.5	5.39
T7	45	0.5	1.5	2.20
T8	45	1.0	0.5	3.82
T9	45	1.5	1.0	6.31
Kij	9.45	6.21	11.0	
	10.8	10.3	11.7	
	12.3	16.1	9.87	
kij	3.15	2.07	3.68	
	3.61	3.44	3.90	
	4.11	5.36	3.29	
R	0.497	1.37	0.393	
Excellent level	A3	B3	C2	
Primary and secondary factors	B > C > A			
Optimal combination	A3B3C2			

**Table 3:** Significant analysis of FO membrane concentration.

Serial Number	Factors			Water Flux (L/m^2^·h)
	A	B	C	
1	1	1	1	1.84
2	1	2	2	3.23
3	1	3	3	4.39
4	2	1	2	2.17
5	2	2	3	3.28
6	2	3	1	5.39
7	3	1	3	2.20
8	3	2	1	3.82
9	3	3	2	6.31
T1	9.45	6.21	11.0	
T2	10.8	10.3	11. 7	
T3	12.3	16.1	9.87	
t1	3.15	2.07	3.68	
t2	3.61	3.44	3.90	
t3	4.11	5.36	3.29	
T	32.6			
Total sum of squares	18.5			
Factor sum of squares	1.37	16.4	0.58	
Error sum of squares	0.19			
Total degrees of freedom	8			
A degrees of freedom	2			
B degrees of freedom	2			
C degrees of freedom	2			
Error degrees of freedom	2			
Factors	SS	df	MS	F
A	1.37	2	0.69	7.25
B	16.4	2	8.19	86.5
C	0.58	2	0.29	3.04
Error	0.19	2	0.09	
Sum	18.5	8		

**Table 4:** Changes in basic properties of FO membranes before and after cleaning.

Parameters	FO Membrane	Value
Water permeability coefficient, A (L·m^−2^·h^−1^·bar^−1^)	After pollution	0.658 ± 0.10
	After cleaning	1.37 ± 0.22
Salt permeability coefficient, B (L·m^−2^·h^−1^·bar^−1^)	After pollution	0.55 ± 0.09
	After cleaning	2.52 ± 0.71
Membrane structure parameters, S (µm)	After pollution	536 ± 41
	After cleaning	475 ± 19

**Table 5:** Cost analysis of diverse cleaning method of FO membrane.

Cleaning Method	Average Flux	Membrane Flux Recovery Rate	Unit Membrane Cleaning Cost
	(L/m^3^·h)	(%)	(Yuan)
Initial	18.3	-	-
Osmotic reflux	11.4	62.3	12
Pressure reflux	12	65.6	24
Cross-flow cleaning	13.5	73.8	14
Acid–base cleaning	15.3	83.6	19

## Data Availability

Not applicable.
